# Interfamilial transmission of HTLV-1 in the South of Bahia, Brazil

**DOI:** 10.1186/1742-4690-11-S1-P50

**Published:** 2014-01-07

**Authors:** Sandra R Gadelha, Milena M Aleluia, Marco AG Mello, Filipe FA Rego, Lucas S Pereira, Bernardo Galvão-Castro, Marilda Gonçalves, Sandra MB Sousa, Luiz C Alcântara

**Affiliations:** 1 LASP/CPqGM/FIOCRUZ, Brazil; 2 Universidade Estadual de Santa Cruz, Ilhéus, Bahia, Brazil; 3 Escola Bahiana de Medicina e Saúde Pública, Brazil; 4 Universidade Estadual do Suoeste da Bahia, Vitoria da Conquista, Bahia, Brazil

## 

The objective of this study was to investigate the prevalence of HTLV in the south of Bahia and to verify the interfamilial transmission of this virus. Initially, blood was collected from pregnant women in hospitals in southern Bahia.The HTLV-1 status was analyzed by ELISA and confirmed by Western Blot and PCR. The HTLV positive women were contacted and visited. In this opportunity, it was explained the results and collected samples from family members. They signed a consent informed and it was applied questionnaire. A total of 2766 pregnant women were analyzed. The prevalence was 1.05% (n=29) (CI95%:0,70-1,50). Besides, it was possible to follow 18 mothers (16 positives and 2 indeterminates) for 2 years. During the following, it was collected samples from 34 family members, including: partners (n=8), mothers (n=7), father (n=1), sisters (n=2), brothers (n=4), sons (n=5), daughters (n=4), others (n=3). Specific reactivity was observed in 11/34 (32,35%) individuals, of which two samples were from children (1 son and 1 daughter - 2,3 and 8 years old, respectively). The others members were: 3 mothers, 1 father, 3 partners, 2 sisters. In one case, we had three generation HTLV-1 positive: grandmother, mother and daughter. These results are very relevant because: (1) so far no studies of HTLV-1 seroprevalence in pregnant/puerperal women from the South region of Bahia had been described, (2) previous studies in areas endemic for the HTLV in Brazil found: 0.84% - Salvador, Bahia and 1.1% in Belo Horizonte, Minas Gerais, (3) evidence of important interfamilial transmission in this area.

